# Who Benefits from An Intervention Program on Foundational Skills for Handwriting Addressed to Kindergarten Children and First Graders?

**DOI:** 10.3390/ijerph17062166

**Published:** 2020-03-24

**Authors:** Livia Taverna, Marta Tremolada, Liliana Dozza, Renata Zanin Scaratti, Domahs Ulrike, Carlo Lallo, Barbara Tosetto

**Affiliations:** 1 Faculty of Education, Free University of Bozen-Bolzano, 39042 Brixen-Bressanone, Italy; liliana.dozza@unibz.it (L.D.); renata.zanin@unibz.it (R.Z.S.); 2 Department of Developmental Psychology and Socialization, University of Padua, 35100 Padua, Italy; marta.tremolada@unipd.it; 3 Department of Women and Child’s Health, Pediatric Hematology, Oncology and Stem Cell Transplant Center, University of Padua, 35100 Padua, Italy; 4 Institute for German Linguistics, University of Marburg, 35032 Marburg, Germany; domahsu@uni-marburg.de; 5 Department of Law, University of Roma Tre, 00154 Rome, Italy; carlo.lallo@uniroma3.it; 6 Medical School for Health Professions “Claudiana”, 39100 Bozen, Italy; Barbara.Tosetto@claudiana.bz.it

**Keywords:** visual-motor integration, fine motor skills, intervention programs in educational setting

## Abstract

This study examined the effectiveness of a 10-wk intervention program based on occupational therapy principles on visual-motor integration skills and fine motor abilities in kindergartners and first graders. We recruited 55 students tested three times with the Visual-Motor Integration Test (VMI) and Movement Assessment Battery for Children-2 (MABC-2): before the intervention (T1), post-intervention (T2) and one month later (T3). Research findings: Significant improvements were found on VMI between T1 and T2, particularly for kindergartners. Neither group of children demonstrated changes on manual dexterity scores. The present study showed that the intervention program led to different changes in the at-risk of motor impairment group than in the not at-risk children. Results indicated that games and stimulation activities helped children below the 16th percentile over time in the manual dexterity domain. A gender effect was observed, with female children increasing their abilities over time more than male peers. Future research should concentrate on stimulating fine motor skills in hand manipulation and test how these abilities influence graphomotor skills and handwriting over time. Finally, more research is needed to determine the impact of activities and games carried out in educational settings.

## 1. Introduction

Handwriting is defined as a survival skill for school-aged children [1]. In early elementary school, handwriting tasks such as copying and writing to dictation are involved in more than 50% of school time [2,3]. For this reason, handwriting is still considered to be one of the fundamental skills to be taught in the first cycle of education. It is also a functional activity not only for the scholastic context but also for everyday life [4]. Nevertheless, learning to write appears to be a challenge for many children who face literacy, with previous studies estimating a prevalence of writing problems ranging from 5% to 25% of the school population [5]. Although this great variability is in all likelihood attributable to the fact that in the pre-school or early education poor handwriters are still not differentiated from children who have neurodevelopment disorders, it is nevertheless evident that educational institutions need early intervention programs to be able to promote the development of foundational skills contributing to the implementation of writing abilities during the first years of formal instruction. Handwriting is experienced as a difficult activity when children get tired and perceive strain or discomfort, even after a short time of writing. In addition, they may struggle to decipher sloppy written words, get in trouble grasping writing tools, may not be able to write down words as fast as their peers and show difficulties in organizing letters and words on the page, producing messy and inaccurate texts. Frustration derived from these experiences causes refusal to dedicate commitment and energies to writing, demotivates children from practicing the necessary skills to acquire a fluent and efficient handwriting and pushes them to develop the belief that they are not skilled writers [6,7], with long-term consequences on future academic success.

### 1.1. Handwriting and Academic Success

Handwriting is a functional skill for academic success in many ways. Scientific research has shown that writing legibly and quickly is important to stay at the pace of school activities; it is required to take notes, to complete homework and to finish the evaluation tests in time [8]. Students need handwriting to communicate their knowledge, and the appearance of written works influence their academic achievements [9]. Poor quality of written texts and scarce neatness determine lower grades irrespective of the compositional and content quality of papers [6]. Transcription skills (handwriting and spelling) have been found to contribute significantly to compositional fluency (rate of composing) for primary and secondary school children, as well as for junior high students [10]. Furthermore, poorly written letters predict academic performances in reading, phonics, language and math for kindergarten children and first graders [11,12]. Moreover, far-reaching negative effects related to frustration with the writing process may result in low motivation to learn, scarce self-efficacy and avoidance of writing tasks [13].

In attempt to identify children that are at-risk of later school failure for poor handwriting, researchers have tried to establish screening programs to detect children with insufficient school readiness who may benefit from early intervention and support [14,15,16]. Domains included in the school readiness’ construct are language and literacy development, cognition and general knowledge, attitudes towards learning, physical well-being and motor development and social and emotional development [17]. According to previous studies [18,19,20], factors connected with the early prediction of good handwriting legibility in first and second graders are fine motor precision, manual dexterity and visual-motor integration. At different ages, and in relation to different schooling orders, fine motor abilities and visual-perceptive skills are required to realize good writing outputs. In fact, in order to realize the letters of the alphabet, respecting their size and spacing, children have to coordinate very different abilities. The visual component allows children to discriminate forms, recognize their specific characteristics and identify their orientation, while the motor component, if properly developed, allows the realization of a wide range of ordered and sequential movements. 

Legible characters are formed by unambiguous traits composing decipherable graphemes in which relative letters’ heights are correct in order to avoid potential confusion in the decoding phase (e.g., “file”/ “fill” or “cure”/ “curl”). Fine motor control, mastering the use of the hand’s small muscles, as well as motor precision, is required to write legibly and to allow the child to achieve the desired traits. For these reasons, fine motor skills have been considered a necessary component in the acquisition of writing, as well as in its expression. Delays in the development of fine motor skills were considered to be the cause of poor writing in terms of readability and fluency [7,18].

Moreover, studies investigating the relationship between visual-perceptive and writing performance have shown that visual-motor integration is the second predictor of writing performance, with high correlations between oculo-manual coordination and readability [21,22,23,24] or between oculo-manual coordination and fluency [3]. Indeed, visual analysis is required to distinguish similar letters (i.e., b and d or p and q) and homonyms such as fair and fare [22]. Recent works on diverse and multiple samples have shown that children scoring higher on design copy tasks gain more on phonological awareness, mathematic skills and reading [25,26]. Longitudinal studies examining the relationship between visual-motor skills and academic achievement demonstrate that third grade literacy [27], math and spelling scores [28] are predicted on the basis of early eye-hand coordination tasks. These results suggest that academic tasks and school demands require abilities to process visual clues, perceive spatial relations among objects and integrate visual information with fine finger movements to assure successful behaviors in the classroom and academic achievements [20,29]. 

### 1.2. Intervention Programs Duration and Gender Differences

Previous studies on intervention programs planned to offer motor remediation have been reported to adopt different approaches including—among others—sensorimotor techniques, perceptual-motor, motor-learning and cognitive training [30]. Regardless of the type of approach, Hillier found that “what is trained is what is improved, whether it is sensory based or motor skills based” [31]. Ideally, more important than the type of intervention adopted seems to be the amount of time devoted to the intervention. Consistent with the theory of motor learning as put by Zwicker and Harris [32], a minimum amount and frequency of practice is needed to reach a permanent change in the capability of movement. 

Studies on the effectiveness of handwriting intervention programs found improvements in fine motor and visual-motor skills in children receiving direct occupational therapy lasting seven or more months [33,34,35,36]. Nevertheless, research has suggested that even short-term interventions targeting fine motor performances in children of lower elementary school ages can lead to significant changes in individuals with or without disabilities [37,38,39,40,41,42]. Furthermore, short-term interventions can be more easily managed and would offer the possibility to be included also in the educational curriculum to improve handwriting foundational skills in preschool children. However, to our knowledge, previous studies have not yet addressed potential interaction effects of gender with intervention programs and the stability of changes over time, while other aspects of gender differences on writing (i.e., text quality, processes of planning and text structure) are well-documented [43]. For example, gender differences attest that 15%–19% fewer boys than girls achieve the expected standards of writing on leaving UK primary schools aged 11 years [44], consistent with other studies reporting a female advantage in writing [45,46]. Girls perform better than boys in spelling, writing essays and sentence composition measured on the Wechsler Individual Achievement Test–Third Edition [47], with a gap increasing with age during the school years [48]. Moreover, in large sample studies, female children have been found to outperform males in fine motor skills, an important predictor of writing skills, still during preschool years [49,50,51]. On the other hand, research has consistently found a gender gap in children’s visual-spatial skills, with boys outperforming girls [52,53,54,55], and these differences remain stable throughout adulthood, especially for mental rotation tasks, as attested from different meta-analyses studies [56,57]. Visual-spatial skills have important implications for future success and achievement. Previous studies have documented that adolescents with higher visual-spatial skills are more likely to graduate from and participate in science, technology, engineering and mathematics fields (STEM). Moreover, visual-spatial skills have been found to predict this participation over and above mathematics and verbal skills [58]. To counteract a possible effect of males struggling more than girls in learning to write and/or females falling behind males on STEM disciplines, educators and teachers should pay additional attention to interventions and programs that improve writing proficiency and are more likely to reduce this gender gap.

### 1.3. Research Aims and Questions

The present study examined the effectiveness of activities and games to stimulate and improve kindergarten children’s and first graders’ visual-motor integration and manual dexterity skills. Since occupational therapy programs target at-risk children and access to individual therapies is reported to be difficult for a number of reasons, other approaches should be taken into consideration [59]. As a result, this study aimed at determining whether students in normal classroom settings would benefit from games and activities based on occupational and remediation therapy principles to enhance school readiness skills like visuo-motor integration and fine finger movements. 

The research questions that guided the present study were:

(1) Did kindergarteners and first graders improve their visual-motor integration ability and their manual dexterity over time in the course of the intervention when mean scores of the Visual-Motor Integration Test (VMI) and Movement Assessment Battery for Children-2 (MABC-2) assessed at T1, T2 and T3 were compared? We expected that the intervention would impact fine motor skills and visual-motor integration abilities of children during the 10-wk training, improving their performances from T1 to T2. No assumptions were formulated about skills’ stability at the follow-up assessment (T3).
(2) Did students at risk of movement difficulty at the baseline in manual dexterity scores make remarkable progress and demonstrate significantly different rates of improvements compared to not at-risk peers along the timepoint assessments? With respect to this issue, we do not formulate any precise hypotheses, because we still do not know if stimulation training in a classroom setting might be as effective as individual therapy.
(3) Do male and female children react to the intervention differently over time? Based on previous research studies in which girls seem to perform better than boys in fine motor skills and boys being advantaged in visual-spatial tasks compared to girls, we expected that females will improve on manual dexterity scores, while males would perform better on visual-perceptual abilities.

## 2. Materials and Methods 

### 2.1. Participants

The current study involved a sample of 55 children recruited from two different public schools in South Tyrol, a region situated in Northern Italy. Forty-two children attended kindergarten (76.4%), and thirteen (23.6%) were first graders. Unlike other Italian areas, South Tyrolean preschool classrooms offer inter-year groups, including children from three to five years. In order to avoid possible confounding results related to different ages, we have randomly selected children attending the last kindergarten year from 6 different classes of one school district and enrolled first graders from another school (see Table 1). There were no drop-out, and all the families of the children joined the research that had been presented to them by class teachers.

Students’ average age was 5 years and one month (age range: 55-83 months; SD: 7.43). Gender was balanced across groups (X^2^_(1)_ = 2.21, *p* = 0.13, Cramer’s V = 0.20): thirty-one children were male (56.4%), and twenty-four were female (43.6%). No significant age differences were found between males and females within the kindergarteners (F _(1,40)_; *t* = 2.52, *p* = 0.66), whereas among first graders, girls were significantly older than boys (F _(1,11)_; *t* = −2.49, *p* = 0.03). Left-handers were 9.1% of the total sample. 

In terms of socioeconomic status (SES), data about parent’s employment, education and material wealth were collected. Groups were balanced when comparing parents’ employment (kindergarteners’ mothers vs. first graders’ mothers: X^2^_(2)_ = 0.75, *p* = 0.68, Cramer’s V = 0.12; kindergarteners’ fathers vs. first graders’ fathers: X^2^_(3)_ = 2.22, *p* = 0.52, Cramer’s V = 0.21) and education (kindergarteners’ mothers vs. first graders’ mothers: X^2^_(3)_ = 0.82, *p* = 0.84, Cramer’s V = 0.12; kindergarteners’ fathers vs. first graders’ fathers: X^2^_(3)_ = 0.66, *p* = 0.88, Cramer’s V = 0.11). No differences have been found with respect to the average number of children (F _(1,46)_; *t* = −1.15, *p* = 0.25) and type of home (X^2^_(4)_ = 1.93, *p* = 0.74, Cramer’s V = 0.20). 

At the time of the investigation, none of the children was diagnosed with learning disabilities or referred for behavioral problems. Parents gave informed consent prior to their children’s participation in the study. Families had the possibility to withdraw from the study at any time, and the data collected were treated in compliance with the privacy law. The study protocol and procedures were approved by the Research and Ethic Committee of the University of Bozen-Bolzano (Bolzano, Italy).

### 2.2. Procedure and Research Design

The schools were contacted by the research team presenting the objectives of the study and the procedure required. The schools were located on the territory of the Autonomous Province of Bolzano and belonged to the Italian-speaking school district. The children who participated in the research can be considered representative of the local school population, since their scores do not differ significantly from the mean performances of all South Tyrolean children undergoing the school readiness tests named “Mondo delle Parole” (M_(*n* = 55 participants)_ = 9.90, SD = 3.53; M_(*n* = 1063 school children)_ = 9.28, SD = 3.70; *t*_(1008)_ = 1,080; *p* = 0.280, ns).

A quasi-experimental research design was used to investigate the effectiveness of 10 weeks of intervention on children’ visual-motor integration skills and fine motor abilities. Children were tested three times with norm-referenced and standardized measures: pre-intervention (T1), post-intervention (T2) and follow-up (T3) within a 2-wk timeframe of each assessment point. Raw scores of the Beery-Buktenica Developmental Test of Visual-Motor Integration (Beery VMI) [60] and the Movement Assessment Battery for Children-2 (Movement ABC-2) [61] were converted into standard scores provided by the manuals on the basis of the child’s chronological age to determine the performance percentile and determine participants’ risk bands. 

### 2.3. Intervention

Participants received direct, regular, age-appropriate stimulation for eye-hand coordination and fine finger movements with playing activities based on pediatric occupational therapy principles in their schools. The educational activities consisted of short games to be carried out in a small group involving practice with in-hand manipulation, transfer of objects from the palm of the hand to the fingers, dissociation and coordination of fingers’ uses, bimanual coordination, discrimination of forms, figure-background separation and completion of paths and tracks. Each type of activity was proposed in three difficulty levels (low, medium and high) following a pre-established program according to which the grade-ordered variants were experienced from the small groups in rotation. In this way, new games were introduced in a progressively difficult order and lasted the entire session. Commonly used and easily available materials such as scissors, glue, pencils and markers of different thickness, woolen and twine threads, tissue paper, pipe cleaners, colored paper and cardboard of various thickness, small spheres of different sizes and weights, raisins, peanuts, rice and pasta for soups in the shapes of letters of the alphabet were required for activities’ completions. 

Intervention included twenty approximately 60-min sessions implemented twice a week for 10 weeks between October 2016 and December 2017. Each session had a preordained course: (1) small groups were created, and the activity of the day was introduced and explained to each small group by occupational therapists and teachers, (2) children played the games, practicing the activities with support and aid from the educational team and (3) each session ended by putting a stamp in a cardboard prepared for the children where they could see the progress of the work and the program. 

### 2.4. Measures

Visual-Motor Integration Test (VMI). The Beery-Buktenica VMI is a widely used standardized assessment tool to test visual-motor integration, visual discrimination and fine motor coordination skills in individuals from early childhood to adulthood. Age-specific updated norms for ages 2 through 18 and above are provided. The VMI is a highly reliable instrument developed and standardized in the USA on a sample of over 11,000 children [62]. This measure provides a rapid screening of those children who have difficulty in organizing and coordinating visual-perceptual analyses with motor performances. The VMI consists of 27 to-be-copied geometric shapes presented in a progressive order of difficulty. The child is requested to look at the different stimuli and to reproduce them with a pencil on a white paper of the evaluation protocol. The VMI contains two additional tests: a visual perception test and motor coordination test, respectively. The purpose of these two tasks is to provide additional information about the child’s visual discrimination ability and fine finger movement skills involved in the tracing tasks. Previous studies have shown that the Beery-Buktenica test has considerable psychometric validity. The test-retest reliability and that between independent judges show high correlation levels: 0.92 and 0.93 respectively. Visual-Motor Integration Test can rely on a high internal consistency with a Cronbach’s value α = 0.88 [63]. Children graphic performances are scored either with 1 or 0 according to the correspondence between drawings and target figures to be copied. The time required for completing the VMI varies from 15 to 20 min.

Movement Assessment Battery for Children-2 (MABC-2). The Movement ABC-2 is a standardized test aimed at identifying motor delays or impairment in children 3 through 16 years of age. The child’s motor functioning is ascertained through the performances exhibited in eight proposed tasks. The MABC-2 completing activities are organized in three categories: (a) manual dexterity, (b) aiming and catching and (c) balance tasks. Standard scores are provided for each component, as well as for the total score. Originally normed on a representative standardization sample of 1172 children from the United Kingdom [61], the adaptation of the MABC-2 used for the current study provides reference scores also for Italian children [59,64]. In this way, it is possible to compare the motor performance of the tested individual with those of typically developing peers using the interpretation manual. Furthermore, the MABC-2 is equipped with a three-color “traffic light” system to assist the examiner in the scoring procedure. Red zone identifies motor performances below the 5th percentile regarded as having a significant movement difficulty. The amber zone includes scores between the 6th and the 15th percentile and are considered as at-risk of movement impairments. The green zone reflects motor performances above the 16th percentile, indicating tasks’ completion in an age-expected norm. The MABC-2 has good psychometric properties, with a reliability coefficient ranging from 0.73 to 0.84. Internal consistency of the MABC-2 test total score is excellent (Cronbach’s α = 0.90). Subscale values range from 0.80 to 0.88 of the Cronbach’s α. 

For the purposes of the current study, only the first part of the test (manual dexterity scale) was administered, in which fingers’ and hands’ fine motor skills are assessed along three tasks: inserting coins into the piggy bank, threading beads and drawing trails. The manual dexterity tasks assess in terms of speed and accuracy how hands are used to manipulate objects and to handwrite. Inserting coins (MD1) and threading beads (MD2) are two timed tasks requiring to record the seconds needed for test completion. For MD1, a separate assessment of the preferred hand (PH) and of the other hand (OH) is foreseen. The drawing trail task (MD3) is evaluated counting the number of errors made in drawing a line that crosses the preprinted tracks. For the manual dexterity scale, lower scores indicate better performance (e.g., higher speed in task completion and greater accuracy with reduced number of errors). The Movement ABC-2 Test provides standardized age-related ratings for each quantitative performance scores. Children needed on average about 20 min to complete the manual dexterity tasks.

### 2.5. Statistical Analysis Plan

Statistical analyses were performed using the SPSS software package version 25.0 (IBM Corp., Armonk, NY, USA) for Mac-OS. Descriptive statistics indicate means and standard deviations of continuous variables (i.e., age of mothers and fathers), as well as counts and proportions of categorical variables (i.e., participants’ genders, parents’ educational levels, type of employment, number of siblings and home situation). Exploratory data analysis and descriptive statistics were performed to provide basic information about the main study variables and to highlight potential relationships between children’s performances. Results are presented in Table 2 according to school order (kindergarten vs. primary school) and time of assessment (pre-intervention, post-intervention and follow-up). Categorical variables were analyzed with a chi-square test for association to determine a groups’ independence. A value of *p* < 0.05 was considered statistically significant. Associations among study variables were assessed by means of Pearson product-moment correlations to determine the strength and direction of the linear relationship between continuous variables (Pearson’s rho values are summarized in Table 3).

To investigate changes over time in standardized VMI scores and MABC-2 manual dexterity performances of kindergartners and first graders, a series of one-way repeated-measure ANOVAs were performed. There were no outliers in the data, as assessed by an inspection of the dependent variables’ boxplots. The assumption of normality for the VMI and the MABC-2 scores was satisfied for both groups of children, for each timepoint, as assessed by a Shapiro-Wilk’s test (*p* > 0.05), respectively. Mauchly’s test was run to verify if differences between all combinations of levels of the within-subject factor had equal variances. When the assumption of sphericity was violated, and the Mauchly’s test was significant at the 0.05 level, results were interpreted using the Greenhouse-Geisser [65] correction for the one-way repeated-measure ANOVAs. 

The second aim of this study was to understand if there was a two-way interaction between the intervention factors and risk group. Indeed, this research question was aimed at untangling whether the effect of the intervention program depends on a group factor, being more effective for children of the risk group. Children performing at or below the 16th percentile at the baseline on the MABC-2 might improve significantly more in their performance compared to not at-risk peers immediately after the intervention (T2) and at the follow-up (T3). To investigate if the intervention’s effectiveness changed differently over time depending on the group factor, a series of two-way mixed ANOVAs were performed with the risk group as the between-subjects factor and intervention as the within-subjects factor.

The third aim of the current study was to investigate how children genders influenced the effectiveness of the intervention on visual-motor integration and manual dexterity skills over time. A series of one-way repeated-measure ANOVAs were run with intervention as the within subject factor with three levels (pre- and post-intervention and follow-up) and gender as the between subject factor (male/female). Interaction effects were tested and reported in the main findings.

## 3. Results

Test scores, means and standard deviations for the study variables are reported in Table 2 separately for educational institutions (kindergarten and primary school), and bivariate correlations are reported in Table 3.

Beery and Beery [60], in their 6th manual’s edition, showed that the VMI has good construct validity, because all correlations among scales were significant beyond the 0.05 level of confidence since the supplemental tests measures part of what the VMI measures, and results correlate at least moderately well with one another. In the present study, children’s outcomes on the Beery VMI and its subtests indicated that visual-motor integration abilities were at all test phases positively correlated with both visual-perceptual skills and hand-motor coordination. The strength of association ranged from moderate (r = 0.40 to 0.59) to strong (r = 0.60 to 0.79) and has been found to be highly significant (*p* < 0.001).

Results showed that visual-motor integration abilities, visuo-perceptive and motor-coordination skills are negatively associated with manual dexterity scores on MABC-2 over time. This negative linear relationship suggests that higher levels of children’s oculo-manual motor integration is associated with lower levels of time spent for completing fine fingers’ tasks.

### 3.1. Effects of the Intervention on Visual-Motor Integration and Fine Motor Skills over Time

The first aim of this study was to assess the effectiveness of the intervention, investigating changes over time on visual-motor integration skills and fine motor abilities in two different age groups: kindergarten and first grade children. Findings from the one-way repeated measure ANOVA for three timepoints are shown in Table 4 for visual-motor integration performances and in Table 5 for manual dexterity scores.

With regards to visual-motor integration skills, analyses revealed a statistically significant difference in VMI performances during the 10 weeks of intervention. Activities elicited statistically significant changes over time in kindergarten children: F _(2, 74)_ = 12.457, *p* = 0.0001, partial η^2^ = 0.252, as well as in first graders: F _(2, 24)_ = 6.936, *p* = 0.004, partial η^2^ = 0.366, respectively. Post hoc analysis with a Bonferroni correction revealed that visual-motor integration performances increased significantly from pre-intervention (T1) to post-intervention (T2) (*d* = 0.69; intermediate effects size) but not from post-intervention (T2) to follow-up (T3) in the two groups of children. Visual discrimination skills, assessed with the supplemental test of visual perception, increased over time in kindergarten children: F _(2, 74)_ = 32.579, *p* = 0.0001, partial η^2^ = 0.468 and in first graders, F _(2, 24)_ = 10.977, *p* = 0.0001, partial η^2^ = 0.478. The statistically significant changes affect all performances assessed in younger children and between T1-T3 and T2-T3 in the older ones. Graphomotor skills tested with the motor coordination supplemental test of the VMI increased in both age groups over time (kindergarten children: F _(2, 74)_ = 9.552, *p* = 0.0001, partial η^2^ = 0.205 and first graders: F _(2, 24)_ = 7.641, *p* = 0.003, partial η^2^ = 0.389). 

In terms of intervention effectiveness on fine motor skills among kindergarteners and first graders, changes over time on MABC-2 scores have been examined. Results indicated that, within both age groups, finger fluency on preferred hand and other hand did not change significantly over the 10-week intervention. Epsilon (ε) values, calculated according to Greenhouse and Geisser [65], were used to correct the one-way repeated measures ANOVA. Exercises did not lead to any valuable increase of skills from pre-intervention to follow-up on the manual dexterity domain (MD1) of kindergarten participants on preferred (PH) and other hand (OH) (PH: F _(15.051, 20.028)_ = 0.752, *p* = 0.451, partial η^2^ = 0.020; OH: F _(25.295, 38.792)_ = 0.652, *p* = 0.471, partial η^2^ = 0.017) and of first graders (PH: F _(19.128, 16.004)_ = 1.195, *p* = 0.306, partial η^2^= 0.091; OH: F _(2, 24)_ = 2.805, *p* = 0.080, partial η^2^= 0.189), respectively. Threading beads abilities increased only for older children (first graders: F _(2, 24)_ = 10.477, *p* = 0.001, partial η^2^ = 0.466), whereas younger ones became faster only after post-intervention but did not maintain their acquired fluency over time (F _(1.428, 52.827)_ = 1.152, *p* = 0.308, partial η^2^ = 0.030). Drawing trails task revealed an increase of performance, because both groups showed a decrease of errors in absolute numbers over time when completing their target figures within the given paths. However, this skill’s improvement does not reach a statistically significant level (kindergarteners: F _(2, 74)_ = 2.158, *p* = 0.123, partial η^2^ = 0.055; first graders: F _(1.408, 16.901)_ = 0.513, *p* = 0.605, partial η^2^ = 0.041).

### 3.2. Effects of the Intervention on different Risk Band Children

The second goal of the study was to determine whether the intervention changes its effectiveness with regards to children’s risk bands (see Table 6). Of interest, we found a significant interaction between the risk band and time on fine finger dexterity performances (i.e., posting coins with preferred hand and threading beads). The interaction effect shows that different risk groups have different patterns of fine motor performances over time (MD1_PH: F _(1.700, 83.294)_ = 4.334, *p* = 0.021, partial η^2^ = 0.081; MD2: F _(1.476, 72.333)_ = 9.824, *p* = 0.001, partial η^2^ = 0.167). As such, from these results, we might expect that intervention efficacy over time depends on the factor group; i.e., intervention efficacy is observable only in the at-risk group. For this reason, the interaction effects were further investigated in terms of simple effects via multiple contrasts, adjusted for Bonferroni correction.

**Figure 1:**
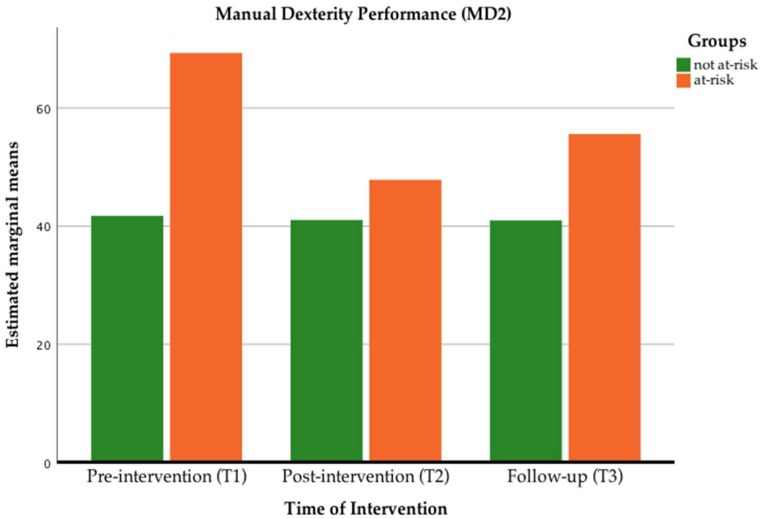
Changes on manual dexterity scores in at-risk and not at-risk children over time.

Figure 1 illustrates different intervention effects in both tested groups. Statistical analysis by means of repeated-measure ANOVAs revealed a main effect of the group in the predicted direction for manual dexterity performance with children included in the at-risk group benefitting from the stimulation program over time (MD1_PH: F _(1, 53)_ = 17.346, *p* = 0.001, partial η^2^ = 0.247 at pre-intervention; F _(1, 50)_ = 2.742, *p* = ns, partial η^2^ = 0.104 at post-intervention; F _(1, 50)_ = 10.697, *p* = 0.002, partial η^2^ = 0.176 at follow-up). Children at-risk for motor difficulty were significantly slower than their peers in completing the posting coins task at the baseline. This difference was no longer statistically significant at the post-intervention assessment, indicating that the stimulation program has reduced the amount of time required to the at-risk group to carry out the task. Moreover, a main effect of intervention in the predicted direction was found for the threading beads motor activity, showing that children experience a significant increase of their performance over time, from baseline to follow-up (MD2: F _(1, 53)_ = 30.555, *p* = 0.001, partial η^2^ = 0.366 at pre-intervention; F _(1, 50)_ = 4.013, *p* = ns, partial η^2^ = 0.074 at post-intervention; F _(1, 50)_ = 15.575, *p* = 0.001, partial η^2^ = 0.238 at follow-up). At baseline, the time measured in seconds needed for threading beads was higher in the at-risk group than in the healthy group (M = 25.5, SE = 4.6, *p* = 0.001). After intervention, the two groups did not show any significant differences in completing the manual dexterity task (M = 6.6, SE = 3.2, *p* = ns). Overall, the intervention with at-risk children showed a very large effect size expressed by the Cohen’s *d* = 1.15 for manual dexterity skills. However, effectiveness of the stimulation program does not seem to result in a long-term improvement, because at the follow-up testing, at-risk children showed again significantly slower performances than their peers (M = 14.6, SE = 3.7, *p* = 0.001).

A main effect of time was found on manual dexterity scores measured in the threading beads activity, showing that significant changes in children’s motor performances occur between pre-intervention to post-intervention and between post-intervention and follow-up. Analyses confirm the visual inspection of the box plots depicted in Figure 1 and reveal that the not at-risk group scores similarly over time, whereas the at-risk group becomes faster after intervention (M diff. T1–T2 = 11.1, SE = 2.5, *p* = 0.001) and worsens its performance when the stimulation of the intervention ceases (M diff. T2–T3 = −3.8, SE = 1.5, *p* = 0.05) but are still significantly faster compared to T1.

### 3.3. Effects of Gender on Visual-Motor Integration and Fine Motor Skills

Findings of the one-way repeated-measure ANOVAs investigating gender and intervention effects on study variables are provided in Table 7.

The ANOVA analysis showed that female children copied more figures than male children and had a higher eye-hand coordination score (F _(1, 49)_ = 6.712, *p* = 0.001, partial η^2^ = 0.120). Furthermore, girls performed better than boys also on the supplemental motor coordination test of the VMI, realizing more figures in the given time (F _(1, 49)_ = 9.082, *p* = 0.004, partial η^2^ = 0.156). 

The two groups of children differed neither on the posting coin activity nor on the drawing trails exercise of the MABC-2 (MD1_PH: F _(1, 49)_ = 3.550, *p* = ns, partial η^2^ = 0.003; MD1_OH: F_(1, 49)_ = 33.726, *p* = ns, partial η^2^ = 0.013). However, girls made fewer mistakes than boys when, realizing the target figure within the trails at each timepoint assessment. There was a statistically significant effect of gender in the threading beads task, with males requiring more time than females to complete the activity (F _(1, 49)_ = 4.665, *p* = 0.036, partial η^2^ = 0.087). 

The intervention effect was significant for several outcome variables. The differences between children’s performances on eye-hand integration and visual discrimination was greater at T2 than at T1 (VMI: F _(2, 98)_ = 21.608, *p* = 0.001, partial η^2^ = 0.306; VMI_MC: F _(2, 98)_ = 10.523, *p* = 0.001, partial η^2^ = 0.177). Follow-up assessments revealed that children improved their scores in all VMI scales when compared to pre-intervention achievements.

## 4. Discussion

Handwriting is the coordination of perceptual, motor and cognitive abilities, which are all involved in the capacity to write letters efficiently [66]. Due to its complexity, it is not surprising that handwriting is prone to disturbances in the course of acquisition. In the present study, we analyzed the effectiveness of an intervention based on occupational therapy principles on handwriting foundational skills in order to enhance school readiness among kindergarteners and first graders. Our aim was to examine children’s improvements on visual-motor integration and manual dexterity skills after stimulation with games and other activities managed in a normal classroom setting. According to previous studies reporting that access to therapies can be difficult for a number of reasons, other approaches need to be considered in which teachers play an important role in supporting children’s development of pre-writing and writing skills. For this purpose, we have proposed a short-term intervention program to improve fine finger movement dexterity and eye-hand coordination abilities in small groups during classroom lessons. To test the effectiveness of the stimulation program, we administered the Beery and Buktenika VMI and the Movement Assessment Battery for Children at three phases (pre-intervention, post-intervention and one month after the end of the activities). Results indicated that children’s visual-perceptive performances and their motor coordination improved from T1 to T2, with some differences related to school order. Kindergarten children displayed significant improvements in coping figures and discriminating geometric targets but did not enhance significantly their ability to control fine movements in reproducing figures while connecting dots within trails. Intervention was effective among first graders on visual-motor integration and motor coordination, but improvements on visual perception did not reach a statistical significance. 

At the fine motor control level, younger and older children did not score higher after the stimulation program. A possible explanation for this finding may be that the planned activities were not structured according to the specific needs of the children or to their already achieved level of competence. Games that were not modulated with respect to the level of individual competence may not have sufficiently motivated the children in carrying out the tasks. Moreover, the short time interval may have led to a lower engagement in the realization of the MABC-2 subtests. However, looking at the raw scores, children’s finger motor precision represented by the ability to recreate a predetermined model within trails or the bilateral hand coordination in inserting beads improves slightly over time, even when not reaching statistical significance levels. Indeed, time required and number of errors to complete these tasks decrease progressively from T1 to T2 for both groups of children. Concerning finger dexterity of the preferred and the other hand, findings suggest that the intervention did not enhance considerably children’s ability to realize fluently the posting coin task, though conclusions should be drawn with caution, because mean differences were calculated in seconds and not in milliseconds, which could have allowed a greater precision in the analysis of performance differences. However, our findings are consistent with previous studies reporting descriptive characteristics of MABC-2 motor tasks in five and six-year-olds [67] with finger fluency and motor precision scores comparable in and across groups. 

Findings of our study should also be interpreted in light of what has been reported in recently published systematic analyses on motor intervention programs for school-aged children [68,69]. In line with previous studies [37,70] designed to develop fine motor control in typically developing children aged 5–6 years, our study had a duration of 10 weeks. However, despite the fact the above-mentioned studies indicate an effectiveness in a 10-wk intervention, the number of hours devoted in each training were slightly different. Indeed, Axford and colleagues [70] designed an intervention program lasting 22.5 h to target motor skills used in daily tasks based on 30-min daily practices. Findings revealed significant gains in writing proficiency, cutting abilities and drawing skills, but no improvements in visual-motor integration was reached, since the mean number of features drawn in the pre- and post-tests were found similar. Diversely, the training of Ohl and colleagues [37] had a total duration of only 5-hrs, with ten 30-min lessons for 10-wks, aiming at strengthening of the intrinsic hand musculature, finger isolation and pincer grasp exercises; separation of the two sides of the hand; translation, rotation, opposition, visual-perceptual and visual-motor skills and bilateral coordination, cutting with scissors, drawing a person, and putting on a coat. They found that five-year-old children improved both on visual-motor integration and on fine motor skills. Given the fact that comparisons with other trainings on the same abilities remains difficult, because based on nonhomogeneous intervention models and on variable amounts of stimulations during the same time interval, we have compared the effect size of our study with those reported in the above-mentioned studies. In line with the study of Axford and colleagues [70], we obtained a moderate clinical effect size (*d* = 0.69) for the significant improvements on visual-motor integration abilities compared to the small effect size reported from Ohl [37]. 

A final concern is the fact that, in the current study, the intervention program led to different changes in a group at-risk of motor impairment compared to not at-risk children. Results indicated that games and stimulation activities helped children classified below the 16th percentile to improve in the manual dexterity domain. Differences in motor performances between children identified as at-risk and their typically developing peers decreased after the intervention program, showing a good remediation chance for children with weaker performances during the stimulation phase. At the follow-up assessment only one month after the end of the activities, at-risk children become slower than their peers in completing motor tasks again, suggesting that fine motor skills have not been consolidated during the stimulation phase and require a longer time of intervention to foster mastery of motor development. If at-risk children are more likely to struggle with everyday school tasks involving fine motor skills [2,4,71], then we might suppose that sooner or later they will want to evade activities they perceive as disadvantaged, resulting in a loss of confidence and in a lack of interest for schoolwork. Hence, additional attention should be devoted to those students who are more at-risk, with the purpose of allowing them to collect positive school experiences, building self-confidence and school success. In this sense, teachers may play an important role in taking early educational actions to counteract this possible effect. One potential way might be to pursue the stimulation of writing foundational skills with games to be included in daily classroom activities. Doing so, it would be possible to aim at improving children’s skills in schoolwork that is not oriented to the task’s completion in a defined interval but to a pleasant play framework to be conducted in small groups. Moreover, the activities modulated according to progressive difficulty levels allow children to have experiences calibrated according to their already developed skills. However, to our knowledge, no other studies than the present one report the effectiveness of a stimulation training on children at-risk of developing fine motor delays taking place in a collaborative group setting during schooltime, by which we obtained a very large effect size (Cohen’s *d* = 1.18 for VMI scores and *d* = 1.15 for MD scores). 

Finally, in our third strand of analyses, we observed a gender effect on the intervention program, with a stronger increase of abilities in female children compared to male peers. Most interestingly, girls outperformed boys in visual-motor integration and in motor coordination tasks, which is in line with previous findings [71,72,73]. In completing the threading beads activity, male children were found to be slower than female. Our results confirm other studies documenting different developmental trajectories for boys and girls. For example, neuroscientists have found an earlier completion of brain development among girls [74] and a stronger hemispheric asymmetry in males. Diverse brain organization was believed to account for multiple performance differences between males and females [75,76]. In the present study, these inequalities between boys and girls may reflect the fact that females show higher readiness than boys for formal schooling and pre-writing skills. Since teachers can adjust their instructions to pupils’ learning paces, these findings might serve to support the modulation of teaching contents and methods adapted to the individual characteristics and needs of children.

## 5. Conclusions

Single results and conclusions reported in this study should be considered with caution due to several limitations. First, the intervention program was not planned to direct children’s individual areas of weakness (visual construction, motor planning, eye-hand coordination, etc.). However, the lack of individualization has to be viewed in light of the authors’ goals to develop a training and intervention program that could be used in an inclusive group educational setting. Since the amount of practice is important to reach effective outcomes, this method has the undoubted advantage of maximizing the practical intervention while, at the same time, a high level of children’s motivation is maintained. Trainings are not done in a separate learning environment and in a face-to-face setting with a therapist. The effectiveness of the intervention program was distributed over the entire class, while most at-risk children have been stimulated in their own school. Moreover, the intervention program improved necessary abilities of children in the natural setting in which instruction is carried out and was not perceived as a special rehabilitation training and might therefore facilitate a generalization and integration of acquired skills to other school tasks. 

Another limitation of the present study was the lack of a control group to assess the effectiveness of the intervention. Thus, it is not possible to determine whether the observed improvements were due to the stimulation program, to children’s maturation over time or to spontaneous learning. Future studies should compare a treatment group with matched peers receiving either a placebo treatment (i.e., “active group”) or no experimental manipulation (i.e., “passive control group”) to further evaluate changes on children’s pre-writing skills. More research is needed to determine the impact of such activities and games carried out in educational settings. 

Third, the sample size of this study was rather small and divided in a two-school order (i.e., 42 kindergarten children and 13 first graders). This heterogeneous distribution of participants represents a further limitation of our study, because, particularly for the second group, the very few subjects per condition (gender or risk band) solicit to interpret results with great caution. It is therefore recommended for future research to include a larger sample size recruited from a larger geographical area to achieve statistical inference. Moreover, studies including a larger number of boys and girls may help to interpret gender differences in critical skills for handwriting acquisition. In addition, the program should be tested with different clinical pediatric populations to better explain how specific individuals respond to the proposed stimulation.

Finally, future studies should take into account the visual integrity of children to exclude that some known disorder (e.g., uncorrected refractive errors, amblyopia or some other visual pathologies) could affect children’s performances in the completion of visual-motor integration tests interfering directly with visual input, which in turn may impact the processing and outputs. Eventually, it would be prudent to introduce the assessment of children’s visual abilities as one of the exclusion criteria in future researches.

Given our findings, we believe that future research should concentrate on the fine motor skills in hand manipulation and their relation to executive functions. Furthermore, since it appears that the amount of practice determines far-reaching outcomes, we think that future intervention programs should be planned for longer periods of time. Although the amount of time required to learn specific motor skills is largely unknown, preliminary evidence from other studies suggest that more practice for longer periods of time might help in stabilizing changes over time [32]. For this reason, we additionally suggest to test the long-term effects of stimulation training (beyond one month), to ensure that the effectiveness will not diminish or dissolve over time. Finally, teachers should be guided and trained in carrying out such activities and educational programs, so that they could take place on a daily basis and reinforce direct and implicit learning.

## Figures and Tables

**Table 1:** Descriptive statistics of the demographic study variables for kindergarteners and first graders at the baseline.

	Kindergarteners (*n* = 42)		First Graders (*n* = 13)		Total Sample (*n* = 55)	
	*n*	%	*n*	%	*n*	%
Age. year.						
4	8	19.0			8	14.5
5	33	78.6			33	60.0
6	1	2.4	13	100	14	25.5
Gender						
male	26	61.9	5	38.5	31	56.4
female	16	38.1	8	61.5	24	43.6
Mother’s Educational Level						
junior secondary school (0–8 years)	9	24.4	4	33.3	13	26.5
high school (9–13 years)	19	51.3	5	41.6	24	49
university (14–18 years)	8	21.6	3	25.1	11	22.5
post-university (beyond 19 years)	1	2.7			1	2
not reported	5	11.9	1	7.7	6	10.9
Father’s Educational Level						
junior secondary school (0–8 years)	10	27.8	4	36.4	14	29.8
high school (9–13 years)	20	55.5	6	54.6	26	55.3
university (14–18 years)	5	13.9	1	9	6	12.8
post-university (beyond 19 Medical School for Health Professions Claudiana.)	1	2.8			1	2.1
not reported	6	14.3	2	15.4	8	14.5
Employment Mother						
housewife	13	31.0	4	30.8	17	30.9
process worker	12	28.6	3	23.1	15	27.3
office worker	10	23.8	5	38.5	15	27.3
professional						
not reported	7	16.7	1	7.7	8	14.5
Employment Father						
unemployed	2	4.8			2	3.6
process worker	18	42.9	8	61.5	26	47.3
office worker	14	33.3	4	30.8	18	32.7
professional	3	7.1			3	5.5
not reported	5	11.9	1	7.7	6	10.9
Home situation						
rent flat	14	33.3	5	38.5	19	34.5
own flat	15	35.7	6	46.2	21	38.2
rent house	2	4.8	1	7.7	3	5.5
own house	2	4.8			2	3.6
other	3	7.1			3	5.5
not reported	6	14.3	1	7.7	7	12.7
Number of siblings						
no sibling	7	16.7	3	23.1	10	18.2
1 sibling	21	50.0	5	38.5	26	47.3
2 siblings	7	16.7	2	15.4	9	16.4
≥3 siblings	1	2.4	2	15.4	3	5.4
not reported	6	14.3	1	7.7	7	12.7
Mother’s age (Mean; SD)	36.0	5.35	39.42	4.87	36.84	5.40
Father’s age (Mean; SD)	39.76	6.39	42.50	5.83	40.43	6.31

**Table 2:** Means and SD of the raw performance scores of the Visual-Motor Integration Test (VMI) and Movement Assessment Battery for Children-2 (MABC-2) assessed at the pre-intervention (T1), post-intervention (T2) and follow-up (T3).

		Kindergarteners (*n* = 42)		First Graders (*n* = 13)	
		M (SD)	Range	M (SD)	Range
*Pre-Intervention*	VMI (max. = 27)				
	Visual-Motor Integration	11.37 (1.97)	8–17	14.77 (2.83)	10–19
	VMI_Visual Perception	11.69 (3.72)	1–19	16.08 (4.09)	10–23
	VMI_Motor Coordination	12.83 (2.76)	6–19	18.08 (3.12)	9–22
					
	MABC-2				
	Manual Dexterity 1 (posting coins) PH	18.14 (6.62)	4–30	16.62 (1.60)	14–19
	Manual Dexterity 1 (posting coins) OH	20.10 (8.82)	1–40	19.23 (2.45)	15–23
	Manual Dexterity 2 (threading beads)	47.12 (18.04)	14–94	40.62 (7.71)	31–57
	Manual Dexterity 3 (drawing trails)	2.57 (2.52)	0–9	0.62 (1.19)	0–4
*Post-Intervention*	VMI (max. = 27)				
	Visual-Motor Integration	12.66 (2.49)	9–19	16.46 (2.47)	12–20
	VMI_Visual Perception	14.50 (3.58)	7–23	18.00 (3.16)	13–25
	VMI_Motor Coordination	12.89 (3.22)	5–19	20.31 (2.86)	14–25
					
	MABC-2				
	Manual Dexterity 1 (posting coins) PH	18.82 (3.76)	9–27	17.46 (2.53)	14–22
	Manual Dexterity 1 (posting coins) OH	21.03 (3.99)	9–30	22.92 (5.88)	17–37
	Manual Dexterity 2 (threading beads)	44.36 (7.66)	28–62	35.31 (10.55)	22–60
	Manual Dexterity 3 (drawing trails)	1.95 (2.11)	0–7	0.62 (0.65)	0–2
*Follow-up*	VMI (max. = 27)				
	Visual-Motor Integration	13.10 (2.11)	9–18	16.85 (2.82)	13–22
	VMI_Visual Perception	16.40 (2.65)	11–22	20.85 (3.21)	15–26
	VMI_Motor Coordination	14.63 (3.15)	6–20	19.85 (2.44)	14–23
					
	MABC-2				
	Manual Dexterity 1 (posting coins) PH	19.00 (2.62)	15–27	18.54 (4.99)	13–31
	Manual Dexterity 1 (posting coins) OH	21.26 (3.21)	15–30	22.00 (6.59)	15–38
	Manual Dexterity 2 (threading beads)	46.74 (9.21)	35–70	32.15 (8.27)	21–51
	Manual Dexterity 3 (drawing trails)	1.77 (1.61)	0–6	0.31 (0.63)	0–2

Note: PH: preferred hand; OH: other hand.

**Table 3:** Bivariate Pearson correlations among study variables for participants (*n* = 55) at pre-intervention (T1), post-intervention (T2) and follow-up (T3).

		Pre-Intervention T1						Post-Intervention T2								Follow-up T3						
		1a	2a	3a	4a	5a	6a	7a	*1b*	*2b*	*3b*	*4b*	*5b*	*6b*	*7b*	1c	2c	3c	4c	5c	6c	7c
*Pre-intervention T1*	1a	-	0.64 ***	0.62 ***	−0.37 **	−0.31 *	−0.43 ***	−0.52 ***	0.67 ***	0.47 ***	0.70 ***	−0.35 **	*−0.19*	−0.47 ***	−0.35 **	0.72 ***	0.55 ***	0.64 ***	**−0.27**	**−0.15**	−0.60 ***	−0.28 *
	2a		-	0.59 ***	−0.42 ***	−0.35 **	−0.48 ***	−0.59 ***	0.56 ***	0.44 ***	0.54 ***	−0.39 **	*−0.19*	−0.44 ***	−0.30 *	0.56 ***	0.56 ***	0.48 ***	**−0.16**	**−0.03**	−0.30 *	**−0.19**
	3a			-	−0.23	−0.16	−0.35 **	−0.53 ***	0.64 ***	0.57 ***	0.78 ***	−0.35 *	*−0.09*	−0.53 ***	−0.44 ***	0.69 ***	0.72 ***	0.73 ***	**−0.14**	**−0.01**	−0.62 ***	−0.48 ***
	4a				-	0.66 ***	0.72 ***	0.44 ***	*−0.20*	*−0.23*	*−0.25*	0.40 **	*0.27*	*0.21*	*0.27*	**−0.19**	**−0.25**	−0.28 *	**0.10**	**0.10**	**0.22**	**0.13**
	5a					-	0.61 ***	0.38 **	*−0.19*	*−0.12*	*−0.23*	0.40 **	0.44 ***	0.30 *	*0.17*	**−0.20**	**−0.12**	**−0.26**	**0.03**	**0.07**	**0.16**	**−0.03**
	6a						-	0.46 ***	*−0.26*	*−0.23*	−0.36 **	0.29 *	*0.20*	0.40 **	0.27 *	−0.35 **	−0.28 *	−0.38 **	**0.16**	**0.19**	0.42 **	**0.04**
	7a							-	−0.60 ***	−0.40 **	−0.61 ***	0.39 **	*0.22*	0.33 *	0.53 ***	−0.43 ***	−0.53 ***	−0.58 ***	**0.21**	**0.00**	0.32 *	0.39 **
*Post−intervention T2*	1b								*-*	0.56 ***	0.71 ***	*−0.26*	*−0.15*	−0.42 **	−0.48 ***	0.74 ***	0.64 ***	0.75 ***	**−0.18**	**−0.10**	−0.46 ***	−0.40 **
	2b									*-*	0.63 ***	−0.28 *	*−0.02*	−0.29 *	*−0.18*	0.50 ***	0.60 ***	0.53 ***	**−0.21**	**−0.09**	−0.49 ***	−0.46 ***
	3b										*-*	−0.34 *	*−0.07*	−0.43 ***	−0.47 ***	0.68 ***	0.62 ***	0.78 ***	−0.32 *	**−0.15**	−0.63 ***	−0.51 ***
	4b											*-*	0.60 ***	0.43 ***	*0.18*	−0.37 **	−0.29 *	−0.29 *	**0.20**	**0.06**	**0.19**	**0.25**
	5b												*-*	*0.11*	*−0.00*	**−0.14**	**−0.13**	**−0.09**	0.37 **	0.45 ***	**−0.03**	**−0.01**
	6b													*-*	0.27 *	−0.45 ***	−0.38 **	−0.47 ***	**−0.13**	**−0.16**	0.66 ***	0.28 *
	7b														*-*	−0.41 **	−0.28 *	−0.59 ***	**0.16**	**0.06**	0.29 *	0.43 ***
*Follow-up T3*	1c															**−**	0.60 ***	0.73 ***	**−0.11**	**−0.03**	−0.57 ***	**−0.26**
	2c																**-**	0.66 ***	**−0.12**	**0.05**	−0.55 ***	−0.32 *
	3c																	**-**	**−0.10**	**−0.07**	−0.60 ***	−0.41 **
	4c																		**-**	0.77 ***	**0.18**	0.29 *
	5c																			**−**	**0.06**	**0.06**
	6c																				**−**	0.28 *
	7c																					-

* *p* < 0.05; ** *p* < 0.01; *** *p* < 0.001. Note: VMI = Visual-Motor Integration Test; VMI_P = Supplemental Test VMI Perception; VMI_MC = Supplemental Test VMI Motor Coordination; MD1_PH = Manual Dexterity 1 Preferred Hand (posting coins); MD1_OH = Manual Dexterity 1 Other Hand (posting coins); MD2 = Manual Dexterity 2 (threading beads); MD3 = Manual Dexterity 3 (drawing trails). Font styles indicate different timepoints for data acquisition: regular = pre-intervention T1; *italic* = post-intervention T2; **bold** = follow-up T3. 1a = VMI; 2a = VMI_VP; 3a = VMI_MC; 4a = MD1_PH; 5a = MD1_OH; 6a = MD2; 7a = MD3. *1b* = VMI; *2b* = VMI_VP; *3b* = VMI_MC; *4b* = MD1_PH; *5b* = MD1_OH; *6b* = MD2; *7b* = MD3. **1c** = VMI; **2c** = VMI_VP; **3c** = VMI_MC; **4c** = MD1_PH; **5c** = MD1_OH; **6c** = MD2; **7c** = MD3.

**Table 4:** Changes over time in kindergartners’ and first graders’ raw scores at the Beery Buktenica Visual-Motor Integration Test measured at T1, T2 and T3.

Groups and Scales	Time of Administration						Statistics					
	Pre-Intervention (T1)		Post-Intervention (T2)		Follow-Up (T3)		F	*p*	ηp^2^	Comparisons	M. Diff.	*p*
	M	SD	M	SD	M	SD						
*Kindergartners’ VMI scores (max. = 27)*												
Visual-Motor Integration	11.37	1.97	12.66	2.49	13.03	2.06	12.457	0.0001	0.252	T1 vs. T2	−1.289	0.005
										T2 vs. T3	−0.368	ns
										T1 vs. T3	−1.658	0.001
Visual Perception	11.47	3.77	14.50	3.58	16.39	2.70	32.579	0.0001	0.468	T1 vs. T2	−3.026	0.001
										T2 vs. T3	−1.895	0.003
										T1 vs. T3	−4.921	0.001
Motor Coordination	12.82	2.90	12.89	3.22	14.61	3.22	9.552	0.001	0.205	T1 vs. T2	−0.079	ns
										T2 vs. T3	−1.711	0.001
										T1 vs. T3	−1.789	0.002
*First grades’ VrMI scores (max. = 27)*												
Visual-Motor Integration	14.77	2.83	16.46	2.47	16.85	2.82	6.936	0.004	0.366	T1 vs. T2	−1.692	0.048
										T2 vs. T3	−0.385	ns
										T1 vs. T3	−2.077	0.029
Visual Perception	16.08	4.09	18.00	3.16	20.85	3.21	10.977	0.001	0.478	T1 vs. T2	−1.923	ns
										T2 vs. T3	−2.846	0.012
										T1 vs. T3	−4.769	0.003
												
Motor Coordination	18.08	3.12	20.31	2.86	19.85	2.44	7.641	0.003	0.389	T1 vs. T2	−2.231	0.008
										T2 vs. T3	0.462	ns
										T1 vs. T3	−1.769	0.024

**Table 5:** Changes over time in kindergartners’ and first graders’ motor performances reported as raw scores at the manual dexterity tasks of the MABC-2 at T1, T2 and T3.

Groups and Scales	Time of Administration						Statistics					
	Pre-Intervention (T1)		Post-Intervention (T2)		Follow-up (T3)		F	*p*	ηp^2^	Comparisons	M. Diff.	*p*
	M	SD	M	SD	M	SD						
*Kindergartners’ MABC-2 scores*												
Manual dexterity 1 PH	17.92	6.47	18.71	3.75	19.03	2.65	0.752	0.451	0.020	T1 vs. T2	−0.789	ns
										T2 vs. T3	−0.316	ns
										T1 vs. T3	−1.105	ns
												
Manual dexterity 1 OH	20.00	8.90	20.95	4.02	21.32	3.23	0.652	0.471	0.017	T1 vs. T2	−0.947	ns
										T2 vs. T3	−0.368	ns
										T1 vs. T3	−1.316	ns
												
Manual dexterity 2	47.82	18.02	44.32	7.76	46.92	9.27	1.152	0.308	0.030	T1 vs. T2	3.500	ns
										T2 vs. T3	−2.605	ns
										T1 vs. T3	0.895	ns
												
Manual dexterity 3	2.45	2.29	1.89	2.11	1.74	1.62	2.158	0.123	0.055	T1 vs. T2	0.553	ns
										T2 vs. T3	0.158	ns
										T1 vs. T3	0.711	ns
*First graders’ MABC-2 scores*												
Manual dexterity 1 PH	16.62	1.60	17.46	2.53	18.54	4.99	1.195	0.306	0.091	T1 vs. T2	−0.846	ns
										T2 vs. T3	−1.077	ns
										T1 vs. T3	−1.923	ns
												
Manual dexterity 1 OH	19.23	2.45	22.92	5.88	22.00	6.59	2.805	0.080	0.189	T1 vs. T2	−3.692	ns
										T2 vs. T3	−0.923	ns
										T1 vs. T3	−2.769	ns
												
Manual dexterity 2	40.62	7.71	35.31	10.55	32.15	8.27	10.477	0.001	0.466	T1 vs. T2	5.308	ns
										T2 vs. T3	3.154	ns
										T1 vs. T3	8.462	0.003
												
Manual dexterity 3	0.62	1.19	0.62	0.65	0.31	0.63	0.513	0.605	0.041	T1 vs. T2	0.000	ns
										T2 vs. T3	0.308	ns
										T1 vs. T3	0.308	ns
												

Note: PH = preferred hand; OH = other hand.

**Table 6:** Means and SD for the main outcome variables and partial eta-squared values associated with risk bands and time-intervention factors.

Outcome Variables	Factors				Statistics									Comparison
	Risk Band	Intervention			Risk			Intervention			Risk by Intervention			
		M (SD) at T1	M (SD) at T2	M (SD) at T3	F	ηp^2^	*p*	F	ηp^2^	*p*	F	ηp^2^	*p*	
VMI					10.609	0.178	0.002 **	12.685	0.206	0.001 ***	0.142	0.003	0.868	T2 > T1 **
	At-Risk	9.78 (1.64)	11.33 (1.58)	11.89 (1.83)										T3 > T1 ***
	Not at-risk	12.76 (2.54)	14.12 (2.98)	14.45 (2.78)										NR > R **
VMI_P														T2 > T1 *
	At-Risk	9.00 (5.09)	10.89 (2.02)	14.78 (2.22)	19.300	0.283	0.001 ***	29.087	0.372	0.001 ***	1.198	0.024	0.306	T3 > T2 ***
	Not at-risk	13.43 (3.72)	16.36 (3.34)	18.12 (3.36)										T3 > T1 *** NR > R ***
VMI_MC														T3 > T2 **
	At-Risk	10.56 (2.69)	9.56 (2.06)	12.11 (3.29)	19.561	0.285	0.001 ***	7.552	0.134	0.001 ***	2.242	0.044	0.112	T3 > T1 **
	Not at-risk	14.93 (3.48)	15.90 (4.07)	16.76 (3.40)										NR > R ***
MD1_PH														
	At-Risk	24.00 (4.40)	19.88 (5.13)	22.13 (2.53)	20.712	0.297	0.001 ***	0.963	0.019	0.385	4.334	0.081	0.021 **	R > NR ***
	Not at-risk	16.40 (5.05)	18.12 (3.11)	18.30 (3.15)										
MD1_OH														
	At-Risk	25.88 (11.39)	22.13 (4.39)	24.50 (4.03)	6.583	0.118	0.013 *	0.283	0.006	0.673	2.968	0.057	0.076	R > NR *
	Not at-risk	18.67 (6.45)	21.33 (4.65)	20.93 (4.12)										
MD2														T2 < T1 ***
	At-Risk	69.25 (17.56)	47.75 (9.86)	55.50 (8.38)	28.218	0.365	0.001 ***	11.316	0.188	0.001 ***	9.824	0.167	0.001 ***	T3 > T2 *
	Not at-risk	41.65 (11.89)	40.95 (8.94)	40.86 (9.97)										T3 < T1 * R > NR ***
MD3														
	At-Risk	4.50 (3.25)	2.88 (2.41)	2.63 (1.76)	14.822	0.232	0.001 ***	4.877	0.091	0.010 **	2.481	0.048	0.089	T3 < T1 **
	Not at-risk	1.51 (1.62)	1.33 (1.75)	1.14 (1.42)										R > NR ***

Note: VMI = Visual-Motor Integration Test; VMI_P = Supplemental Test VMI Perception; VMI_MC = Supplemental Test VMI Motor Coordination; MD1_PH = Manual Dexterity 1 Preferred Hand (posting coins); MD1_OH = Manual Dexterity 1 Other Hand (posting coins); MD2 = Manual Dexterity 2 (threading beads); MD3 = Manual Dexterity 3 (drawing trails). * *p* < 0.05; ** *p* < 0.01; *** *p* < 0.001.

**Table 7:** Means and SD for the main outcome variables and partial eta-squared values associated with gender and time-intervention factors.

Outcome Variables	Factors				Statistics									Comparison
	Gender	Intervention			Gender			Intervention			Gender by Intervention			
		M (SD) at T1	M (SD) at T2	M (SD) at T3	F	ηp^2^	*p*	F	ηp^2^	*p*	F	ηp^2^	*p*	
VMI					6.712	0.120	0.013 *	21.608	0.306	0.001 ***	2.703	0.052	0.072	T2 > T1 ***
	Male	11.76 (2.43)	12.90 (2.83)	12.93 (2.13)										T3 > T1 ***
	Female	12.86 (2.86)	14.59 (2.95)	15.41 (3.00)										F > M *
VMI_P					3.694	0.070	0.060	42.550	0.465	0.001 ***	0.122	0.002	0.886	T2 > T1 ***
	Male	12.03 (3.85)	14.66 (2.91)	16.69 (2.52)										T3 > T2 ***
	Female	13.45 (4.83)	16.36 (4.57)	18.64 (4.14)										T3 > T1 ***
VMI_MC					9.082	0.156	0.004 **	10.523	0.17	0.001 ***	0.061	0.001	0.941	T3 > T1 ***
	Male	12.97 (3.25)	13.48 (4.12)	14.66 (3.50)										T3 > T2 *
	Female	15.73 (3.80)	16.50 (4.49)	17.64 (3.56)										F > M **
MD1_PH					0.133	0.003	0.717	1.449	0.029	0.240	1.370	0.027	0.258	
	Male	17.13 (6.22)	18.87 (3.82)	18.50 (2.52)										
	Female	18.24 (4.79)	17.71 (2.95)	19.48 84.27)										
MD1_OH					0.659	0.013	0.421	2.172	0.042	0.136	0.402	0.008	0.602	
	Male	19.83 (9.38)	20.80 (4.71)	20.93 (3.46)										
	Female	19.76 (4.77)	22.38 (4.33)	22.29 (5.21)										
MD2					4.665	0.087	0.036 *	2.251	0.044	0.127	0.032	0.001	0.931	M > F *
	Male	48.37 (19.31)	44.27 (8.21)	45.80 (10.59)										
	Female	42.57 (10.01)	38.81 (10.06)	39.38 (10.84)										
MD3					1.160	0.023	0.287	1.971	0.039	0.145	0.474	0.010	0.624	
	Male	2.30 (2.33)	1.73 (1.91)	1.47 (1.38)										
	Female	1.52 (1.99)	1.33 (1.98)	1.24 (1.81)										

Note: VMI = Visual-Motor Integration Test; VMI_P = Supplemental Test VMI Perception; VMI_MC = Supplemental Test VMI Motor Coordination; MD1_PH = Manual Dexterity 1 Preferred Hand (posting coins); MD1_OH = Manual Dexterity 1 Other Hand (posting coins); MD2 = Manual Dexterity 2 (threading beads); MD3= Manual Dexterity 3 (drawing trails). * *p* < 0.05; ** *p* < 0.01; *** *p* < 0.001.
